# Cellular Proteins Associated with the Interior and Exterior of Vesicular Stomatitis Virus Virions

**DOI:** 10.1371/journal.pone.0104688

**Published:** 2014-08-08

**Authors:** Megan Moerdyk-Schauwecker, Sun-Il Hwang, Valery Z. Grdzelishvili

**Affiliations:** 1 Department of Biological Sciences, University of North Carolina at Charlotte, Charlotte, North Carolina, United States of America; 2 Center for Biomedical Engineering and Science, University of North Carolina at Charlotte, Charlotte, North Carolina, United States of America; 3 Proteomics Laboratory for Clinical and Translational Research, Carolinas HealthCare System, Charlotte, North Carolina, United States of America; Lady Davis Institute for Medical Research, Canada

## Abstract

Virus particles (virions) often contain not only virus-encoded but also host-encoded proteins. Some of these host proteins are enclosed within the virion structure, while others, in the case of enveloped viruses, are embedded in the host-derived membrane. While many of these host protein incorporations are likely accidental, some may play a role in virus infectivity, replication and/or immunoreactivity in the next host. Host protein incorporations may be especially important in therapeutic applications where large numbers of virus particles are administered. Vesicular stomatitis virus (VSV) is the prototypic rhabdovirus and a candidate vaccine, gene therapy and oncolytic vector. Using mass spectrometry, we previously examined cell type dependent host protein content of VSV virions using intact (“whole”) virions purified from three cell lines originating from different species. Here we aimed to determine the localization of host proteins within the VSV virions by analyzing: i) whole VSV virions; and ii) whole VSV virions treated with Proteinase K to remove all proteins outside the viral envelope. A total of 257 proteins were identified, with 181 identified in whole virions and 183 identified in Proteinase K treated virions. Most of these proteins have not been previously shown to be associated with VSV. Functional enrichment analysis indicated the most overrepresented categories were proteins associated with vesicles, vesicle-mediated transport and protein localization. Using western blotting, the presence of several host proteins, including some not previously shown in association with VSV (such as Yes1, Prl1 and Ddx3y), was confirmed and their relative quantities in various virion fractions determined. Our study provides a valuable inventory of virion-associated host proteins for further investigation of their roles in the replication cycle, pathogenesis and immunoreactivity of VSV.

## Introduction

Vesicular stomatitis virus (VSV, family *Rhabdoviridae*) is a prototypic nonsegmented negative-strand RNA virus (order *Mononegavirales*) serving as a model for other human and animal pathogens including other rhabdoviruses such as rabies virus. VSV is also being widely investigated as a vector for vaccines (reviewed in [1]), gene therapy (reviewed in [2]) and oncolytic (anticancer) virotherapy (reviewed in [3], [4]). As a result, currently two phase I human clinical trials evaluating the safety of the VSV-based HIV vaccines (Clinicaltrials.gov, trials NCT01438606 and NCT01578889) are currently in progress. In addition, a phase I human clinical trial using replication-competent oncolytic VSV against hepatocellular carcinoma is in progress (trial NCT01628640). VSV replicates in the cytoplasm and contains a nonsegmented, negative-strand RNA genome of approximately 11 kb encoding five viral proteins, all of which are included in the mature virion. The large polymerase protein (L) and phosphoprotein (P) together form the viral RNA dependent RNA polymerase (RdRp). In mature virions, the RdRp is associated with the nucleocaspid (N) protein encapsidated viral genome, together forming the ribonucleoprotein (RNP) complex. The matrix (M) protein is responsible for nucleocapsid condensation and is the primary driving force behind viral budding through the host cell plasma membrane, whereby the virion obtains its lipid bilayer envelope. Embedded in that envelope is the transmembrane glycoprotein (G), which is essential for receptor binding and cell entry (reviewed in [5]).

VSV virions, like those of many viruses, contain not only virus encoded-proteins, but also host (cellular) proteins [45]. Some of these proteins are enclosed within the virion structure, while others, in the case of enveloped viruses like VSV, are embedded in the host-derived membrane [6]. While many of these incorporations could be “accidental”, others may reflect the mechanism of virus assembly, be necessary for viral function, impact host range and infection efficiency, or influence the outcome of subsequent infections. For example, a recent study showed that depletion of a number of host proteins normally incorporated into herpes simplex virus type-1 virions not only reduced virion production in those cells, but that new infections with the resulting virions also yielded fewer progeny virions even in cells expressing normal levels of the host protein [7]. The host-derived proteins of a virus may also affect the host immune response. This is an especially important consideration for viruses, including VSV, used in therapeutic applications where large numbers of virus particles are administered, as it may influence efficacy as well as the potential for adverse side effects. Incorporation of ICAM-I into the envelope of human immunodeficiency virus type-1 (HIV-1) not only increased infection efficiency [8], [9], [10], [11] but also interfered with virus neutralization by host antibodies [12], [13], [14], [15]. In another example, the presence of host complement control proteins such as CD46, CD55 and CD59 in the viral envelope has been shown to protect against antibody dependent complement mediated virus lysis in several viruses including human T cell leukemia/lymphoma virus type I [16], human cytomegalovirus [16], hepatitis C virus [17], HIV-1 [18], [19], extracellular enveloped vaccinia virus [20], simian virus 5 [21] and mumps virus [21].

To comprehensively look at host protein incorporation, a number of purified viruses have been analyzed by mass spectrometry including poxviruses [22], [23], [24], herpesviruses [25], [26], [27], [28], [29], [30], [31], [32], orthomyxoviruses [33], coronaviruses [34], [35], retroviruses [36], [37], [38], [39], paramyxoviruses [40], [41], baculoviruses [42], hytroviruses [43] and arteriviruses [44]. Our previous study examined VSV virions grown in three different cell lines (BHK-21, 4T-1 and A549) originating from different species, and found a number of similarities and differences in the host protein content [45]. Here we conduct an analysis not only of intact (“whole”) virions, but also virions treated with proteinase K (ProK) to remove surface proteins to look at the localization of host protein incorporation into the virion.

## Materials and Methods

### Cells and viruses

Syrian golden hamster kidney fibroblast cells (BHK-21; ATCC# CCL-10) were grown in monolayer cultures maintained in Minimum Essential Medium (Eagle's MEM, Cellgro) supplemented with 9% fetal bovine serum (FBS, Gibco), 0.3% glucose (w/v), 3.4 mM L-glutamine, 90 U/ml penicillin and 90 µg/ml streptomycin, and kept in a 5% CO_2_ atmosphere at 37°C. Infectivity [plaque forming units (PFU) per ml] of virus stocks was determined by standard plaque assay on BHK-21 cells. Recombinant wild-type (wt) VSV (Indiana serotype) was generated previously [46] using pBS-L, pBS-P, pBS-N, and pVSVFL(+) plasmids, and was kindly provided by John K. Rose (Yale University) [47].

### Whole virion purification

To grow and purify virus VSV, BHK-21 cells were infected with at a multiplicity of infection (MOI) of 0.001 and incubated at 37°C in media containing 5% FBS. Virus containing media was collected around 24 hours (h) post infection (p.i.) when most cells were infected but significant cell detachment had not yet occurred in order to maximize exclusion of cellular debris. The media was centrifuged at 3,000× g for 10 minutes (min) to remove large cellular debris. The virus was then purified as described in [48], with slight modifications. In brief, clarified supernatants were underlayed with 5 ml 20% (w/v) sucrose in HEN buffer (10 mM HEPES pH 7.4, 1 mM EDTA, 100 mM NaCl) and centrifuged at 28,000 rpm and 4°C for 3.5 h in a Beckman SW32 Ti rotor. The resulting virus-containing pellet was resuspended overnight in HEPES buffered saline, pH 7.5 [HBS; 21 mM HEPES, 140 mM NaCl, 45 mM KCl, 0.75 mM Na_2_HPO_4_, 0.1% (w/v) dextrose] and then centrifuged in a 7.5–27.5% continuous gradient of Optiprep (Axis Shield) in HBS at 26,500 rpm and 4°C for 30 min using a Beckman SW40 Ti rotor. The virus-containing band was removed from the gradient, diluted with ET buffer (1 mM Tris-HCl pH 7.5, 1 mM EDTA), pelleted by centrifugation at 27,000 rpm and 4°C for 1.5 h using a Beckman SW40 Ti rotor and resuspended in ET buffer.

### Isolation of ProK treated virions and RNP complexes

For protease treatment, purified virions were treated with 0.08 µg ProK per 1 µg total protein, re-purified by centrifugation through a sucrose cushion as previously described [45] and resuspended in ET buffer. For isolation of viral RNP complexes, purified virions not treated with ProK were disrupted as previously described [49]. In brief, virions were disrupted in a buffer containing a final concentration of 10 mM Tris-HCl (pH 8.0), 5% glycerol (v/v), 0.4 M NaCl, 1.85% Triton X-100, and 0.6 mM DTT, and pelleted through a 30% glycerol cushion onto a 100% glycerol cushion by centrifugation at 38,000 rpm and 4°C for 2 h using a TLA 100.2 rotor. Under these conditions, proteins with moderate to high affinity for the viral nucleocapsid, including the viral L/P polymerase complex, remain associated with the nucleocapsid [49]. The recovered RNP complexes were diluted 1∶1 (v/v) with a buffer containing 50 mM Tris-HCl (pH 8.0), 10 mM NaCl, 5 mM MgCl_2_ and 2 mM DTT.

### Electron microscopy

Virions were absorbed to carbon-formvar coated grids (Electron Microscopy Sciences) by floating grids on 5 µl drops of sample for 30 seconds (s). Grids were blotted dry, stained with 2% uranyl acetate in water for 30 s, blotted to remove excess stain and air-dried. Samples were visualized using a JEOL JEM 2100 LaB6 Transmission Electron Microscope.

### Protein identification following 1D-SDS-PAGE

Total protein equaling 50 µg for purified virion samples and 60 µg for ProK treated virions was separated on a 10% Tris-Glycine SDS-PAGE gel under reducing conditions and stained with Coomassie Brilliant Blue R250. These quantities were chosen so that the total amount of viral N plus P protein was approximately equivalent for both sample types. Each gel lane was cut into 13 or 14 gel bands for analysis. Gel pieces were subjected to in-gel trypsin digestion and the resulting peptides were extracted from the gel matrix, separated using Waters ACQUITY ultra performance liquid chromatography (UPLC) with nano-split and analyzed using a LTQ-XL tandem mass spectrometer (ThermoFisher Scientific, Waltham, MA) as described previously [50]. Briefly, samples were separated by a 65 min linear gradient from 95% Solvent I (0.1% formic acid in water)/Solvent II (0.1% formic acid in acetonitrile) to 50% Solvent I/II at a flow rate of 500 nl/min on reversed phase chromatography using a trap/elute method with a in-house C_18_ sample trap in line with a C_18_ analytical column. Each full MS scan was followed by eight MS/MS scans of the most intense ions with data-dependent mode using the dynamic exclusion option (Top 8 method). The spectra were searched using the SEQUEST algorithm of the Bioworks software (ThermoFisher, San Jose, CA; version 3.3.1 SP1) against the IPI.HUMAN.v.3.18 and IPI.MOUSE.v.3.18 databases concatenated with a VSV and Sendai virus protein database. A parent ion mass tolerance of 2.0 Da, fragment ion mass tolerance of 1.0 Da, and a 16 Da differential modification for methionine oxidation were used for search parameters. Protein identifications were accepted when the peptide probability was greater than 95.0% [51], the protein probability was greater than 99.0%, and contained at least two unique peptides. Scaffold software was used for data compiling of each group and calculating spectral counts, unique peptides, percent coverage and emPAI [52], [53], [54]. While a single set of gel bands was isolated for each sample, three replicate UPLC-MS/MS runs were conducted for the whole and ProK virion samples, although one set of runs could not be used for the whole virions due to poor data quality.

### Gene Ontology enrichment analysis

Protein enrichment was analyzed using the Database for Annotation, Visualization and Integrated Discovery (DAVID) v.6.7 [55], [56]. Using the *Mus musculus* genome as the background data set, protein sets were analyzed for enrichment using the terms from the Gene Ontology (GO) biology process, cellular component or molecular functions Fat databases. The GO Fat databases contain the more specific GO terms while excluding the more general terms. The enriched terms were then subjected to cluster analysis using the default settings, to identify groups of related enriched terms, with overall enrichment scores based on the EASE scores of the member terms. The broadest term, representing most if not all the proteins in the cluster, is used here to describe the cluster.

### Immunoblot analysis

Cellular lysates were prepared by mock infecting BHK-21 cells or by infecting them with VSV at a MOI of 0.05. Cells were harvested at 18 h p.i. and lysed in RIPA buffer (25 mM Tris-HCl pH 7.6, 150 mM NaCl, 1% NP-40, 1% Sodium deoxycholate and 0.1% SDS). Protein concentrations of cellular lysates, purified virions, ProK treated virions and RNP complexes were determined by Bradford assay. 50 µg of purified virions, 60 µg ProK treated virions, 15 µg of RNP complexes and 10 µg of cellular lysates were separated on Tris-Glycine 10% SDS-PAGE gels, transferred to PVDF membranes and rapidly stained with the reversible dye Ponceau S prior to the use of antibodies to confirm viral protein loading and the quality of protein transfer from gel to membrane. Membranes were blocked in TBS (0.5 M NaCl, 20 mM Tris pH 7.5) with 0.1% Tween 20 and 5% non-fat milk powder and then probed with antibodies against Cc2d1a (A300-285A; Bethyl Laboratories), Yes1 (3201; Cell Signaling), ITCH (32/Itch; BD Transduction Laboratories), Hsc70 (K-19; Santa Cruz Biotechnology), Prl2 (05-1583; Millipore), Ck1 (2655; Cell Signaling), or Ddx3y (PA5-22050; Thermo Scientific). Detection was with species-specific horseradish peroxidase-conjugated secondary antibodies using the Enhanced Chemiluminescence Plus (ECL+) protein detection system (GE Healthcare) and captured using a ChemiDoc-It imaging system (UVP imaging, Upland CA).

## Results and Discussion

### Purification and treatment of viruses

**Figure 1:**
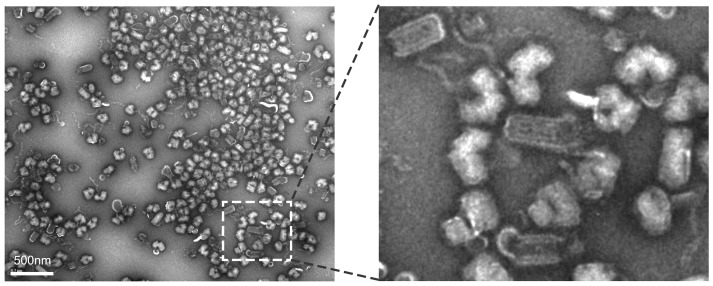
Transmission electron micrographs of purified VSV virions. Virions were absorbed to carbon-formvar coated grids and negatively stained with uranyl acetate. The image on the right is a close-up of the boxed area in the image on the left.

Recombinant wt VSV was grown in BHK-21 cells and purified using gradient centrifugation. The purity of the resulting material was then examined by electron microscopy (EM). As seen in Figure 1, nearly all of the material present was clearly identifiable as VSV virions, although many of them were bent, a previously observed form that is generally believed to be infectious [57] and may represent an EM processing artifact [58], [59]. The titer of these purified virions on BHK-21 cells was 1.4×10^11^ PFU/ml, demonstrating this preparation was highly infectious.

**Figure 2:**
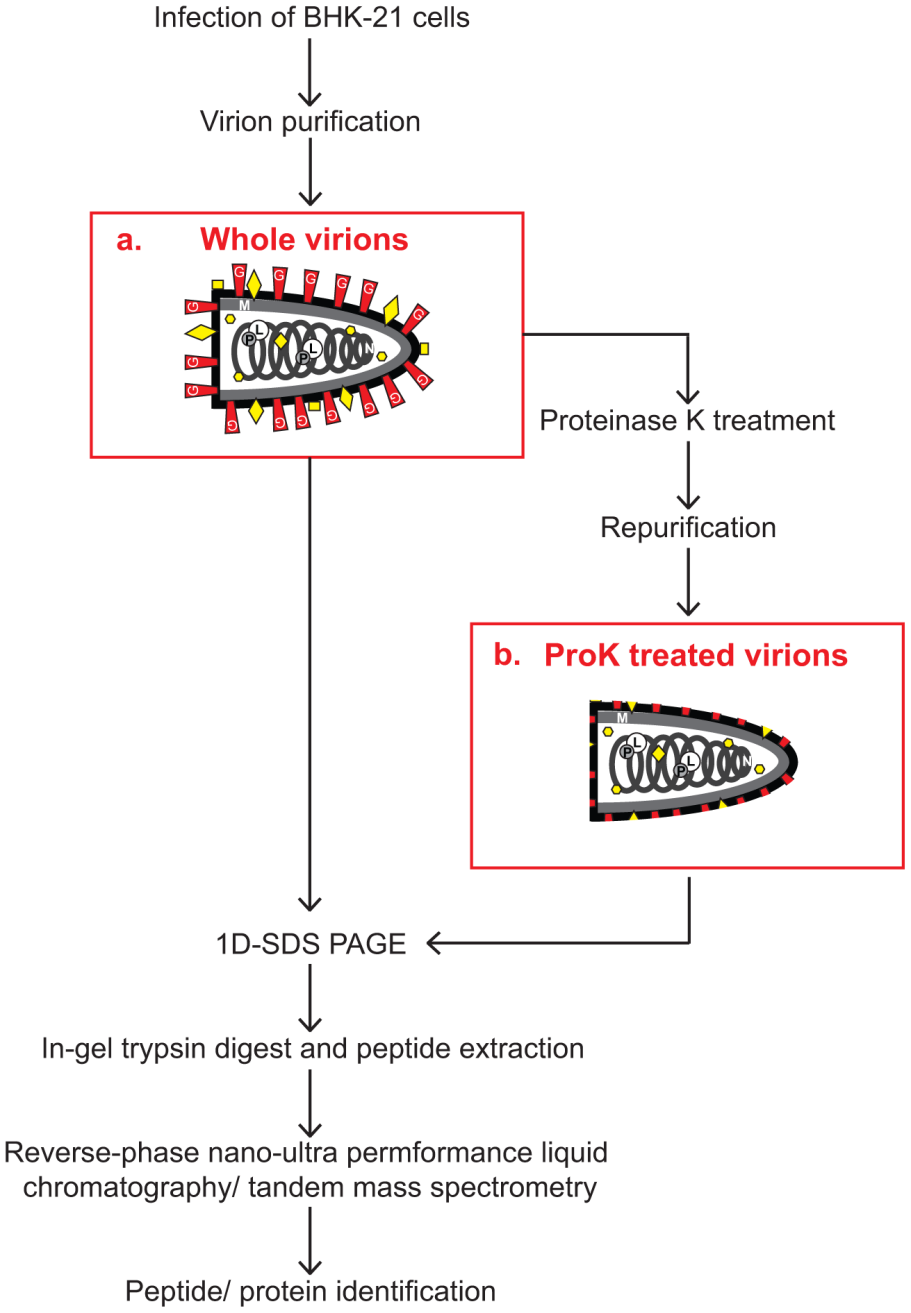
Overview of sample preparation and experimental approach. Mass spectrometry based analysis of host protein content was conducted on (a) purified whole virions and (b) purified virions treated with proteinase K to remove surface proteins. G, glycoprotein; M, matrix protein; P, phosphoprotein; L, large polymerase protein; N, nucleocapsid protein; yellow shapes, host proteins.

A portion of these whole virions was used directly for proteomic analysis and additional confirmation assays, while the other portion was treated with ProK and re-purified (Fig. 2). ProK treatment cleaves all proteins on the exterior of the virions as well as the extracellular domain of all transmembrane proteins. However, it cannot penetrate the viral envelope, leaving all VSV proteins except for G intact, as well as all host proteins contained within the virion and the transmembrane and cytoplasmic domains of the host membrane proteins located in the viral envelope. This treatment also tends to alter the density of any remaining cell derived vesicles relative to the treated virions, facilitating their removal during the subsequent repurification step [60]. However, proteins on the interior of any residual cellular vesicles would still be detectable.

### Identification of virion-associated proteins

**Figure 3:**
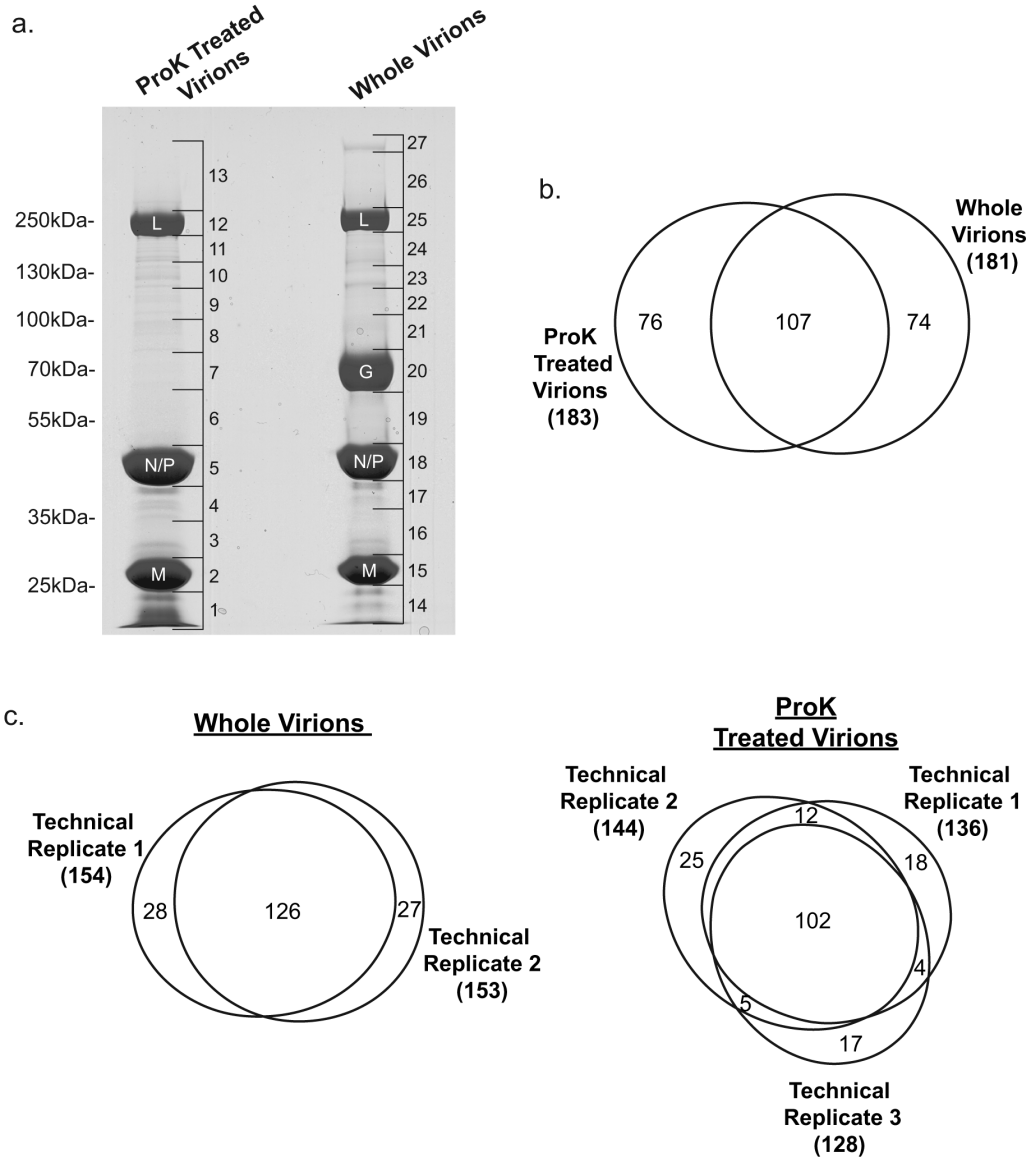
1D SDS-PAGE and protein identification by mass spectrometry. (a) Total protein from whole virions or proteinase K (ProK) treated virions was separated by 1D-SDS-PAGE and stained with Coomassie Brilliant Blue R250. Brackets on the right indicate bands cut out and analyzed by mass spectrometry (MS). The position of the molecular mass markers is indicated on the left. The bands containing the viral large polymerase protein (L), glycoprotein (G), nucleocapsid protein (N), phosphoprotein (P), and matrix protein (M) are indicated. Venn diagrams indicate the number of proteins identified by MS, and the degree of overlap in the proteins identified (b) between sample types and (c and d) between technical replicates of the same sample type.

For mass spectrometry (MS) analysis, total protein from 50 µg purified virions or 60 µg ProK treated virions was separated by 1D-SDS-PAGE. These quantities were chosen so that the amount of viral N and P protein was approximately equal in both sample types as determined by Coomassie staining (Fig. 3a) to facilitate comparisons between samples. Importantly, while N, P and L bands were similar in both virion preparations, no full length G protein was visible for ProK treated virions, indicating the ProK treatment was highly successful. After separation by SDS-PAGE, the resolved proteins were cut out in a series of bands as indicated in Figure 3a. These bands were then subjected to in-gel trypsin digest and the resulting peptides were extracted, separated by UPLC and analyzed by tandem MS (MS/MS). In this study, virions were grown on BHK-21 cells as it is the standard cell line for growth of VSV and the high virion yields aid in purification. However, a complete database of Syrian hamster proteins is not available. Instead, peptides were identified by matching them to either a human or a mouse database. The results from both searches were highly similar; therefore only those from the search of the mouse database are presented here.

In addition to all five viral proteins, 257 different host proteins were identified in total (Table S1), with 181 proteins identified in whole virions and 183 proteins identified in ProK treated virions (Fig. 3b). This is a considerably larger number of host protein than identified in our previous study where 30 proteins were identified in a different preparation of BHK-21 derived virions [45]. This is likely primarily due to dividing the samples into a larger number of bands following 1-D SDS-PAGE. In addition, in the present study we used enhanced sample separation through use of UPLC, which should also allow for more sensitive detection. Of the 30 proteins detected in BHK-21 derived whole virions in our previous study, 27 were detected in at least one sample type here, showing consistency in the proteins detected in independent virion preparations purified using two different methodologies (continuous iodixanol gradient versus discontinuous sucrose gradient). Differences in the purity of the virions prepared using these two methods could also contribute to differences in the number of host proteins identified. However, EM images, staining of total protein on SDS-PAGES gels and infectivity (4.2×10^7^ PFU/µg total protein for the present method versus 2.4×10^7^ PFU/µg total protein for our previously published method [45]) all indicate virions prepared using the method presented here are of equal or greater purity to those used previously.

Surprisingly, more than 40% of proteins found in ProK treated virions were not found in whole virions even though the ProK treated virions were derived from the whole virion preparation (Fig. 2). One possibility is that some of these proteins may have been masked by more abundant host or viral proteins that were removed by treatment. For example, 25% (19/76) of the proteins detected in ProK treated virions but not whole virions were from the position correlating to that of the viral G protein in the whole virion sample (Table S1 and Fig. 3a). Another possible explanation is that these proteins are in low abundance or possess other properties making them difficult to detect consistently by MS. Of the proteins found only in ProK treated virions, 50% of proteins were identified based on detection of only two unique spectra (the minimum number allowed under our criteria), as opposed to 15% for proteins also identified in whole virions (Table S1). Unsurprisingly, ease of detection also seemed to be a major determinate of consistency of detection between technical replicates. Proteins found in all technical replicates were identified based on only two unique spectra in 19% and 17% of cases for whole virions and ProK treated virions respectively, while proteins found in only one technical replicate were identified based on only two spectra in 71% and 77% of cases (Fig. 3c–d and Table S1).

In general, the position of the identified proteins on the gel was consistent with the predicted molecular mass of the proteins (Table S1 and Fig. 3a) although some appeared at higher molecular masses, likely due to post-translational modifications affecting protein mobility. Exceptions to this general pattern were keratins, common environmental contaminants, which were found across a wide range of molecular weight and therefore excluded from this analysis. Following ProK treatment, 74 of the 181 proteins identified in whole virions were no longer detectable, likely due to their removal by the treatment. However, other proteins were still detected in the ProK treated sample but appeared at a lower molecular mass versus the predicted molecular mass and/or that observed for whole virions, likely due to the removal of the extracellular domain. For example, hepatocyte growth factor receptor was found in a slice spanning approximately 140–225 kDa in the whole virion while it was found in slices spanning approximately 50–80 kDa in ProK treated virions. This demonstrates the value of information about the approximate size of the proteins in the sample (as determined by the position of the gel band) in interpreting data from proteinase treated samples.

**Figure 4:**
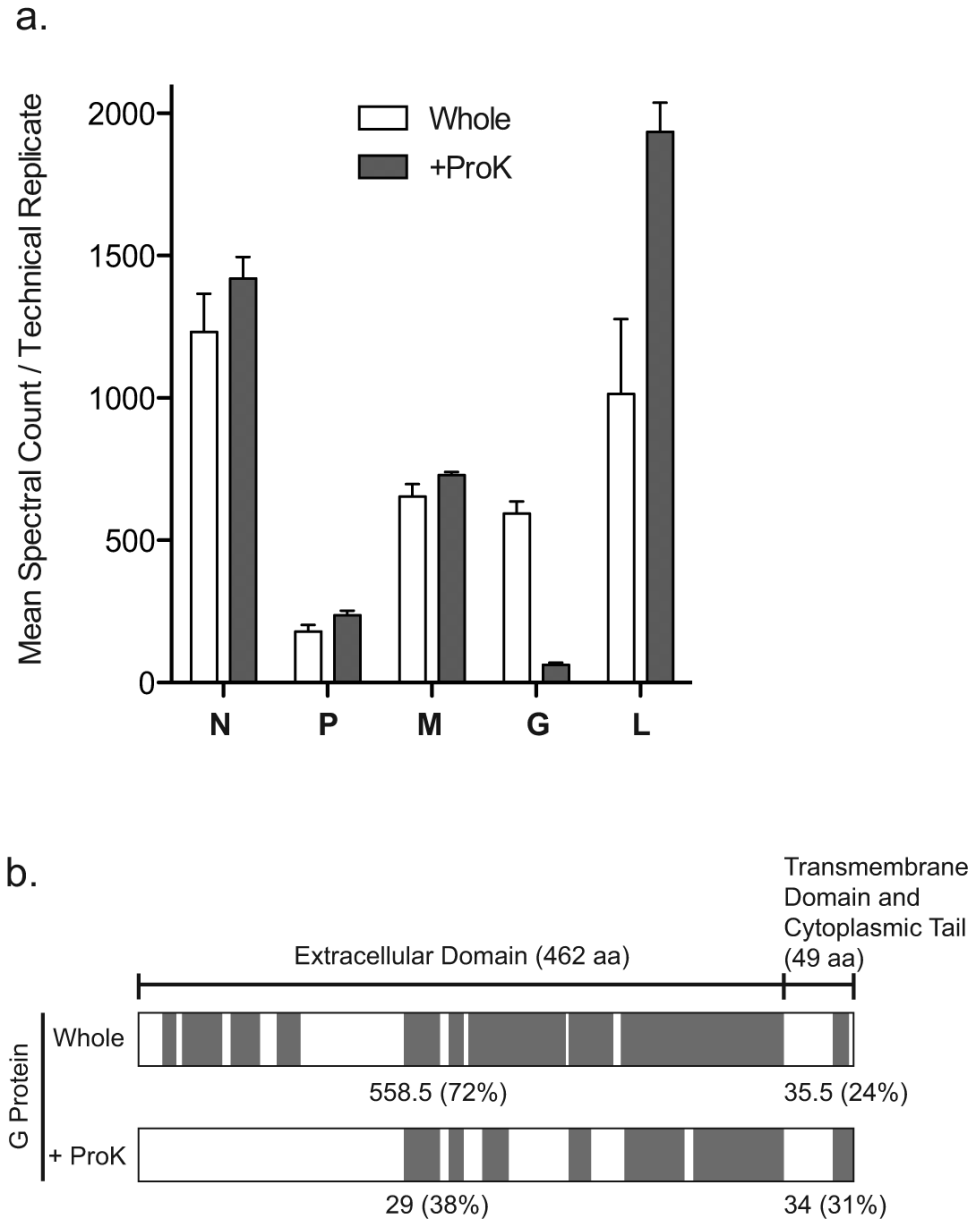
Detection of VSV proteins by mass spectrometry. (a) Mean spectral counts per technical replicate for VSV encoded proteins detected in both whole virions and proteinase K (ProK) treated virions. (b) Location of VSV G protein peptides identified in both whole and ProK treated virions as indicated by shaded boxes. Values given below the diagrams indicate the mean spectral counts per technical replicate for the indicated domain(s) with the overall percent sequence coverage given in parentheses.

Although the primary focus of this study was determining host protein incorporation and localization in VSV virions, our search database also included VSV protein sequences allowing for their detection. In untreated samples, a mean of 1231-182 spectra per technical replicate were detected for each of the five VSV encoded proteins (Fig. 4a). The number of spectra went up upon ProK treatment for all VSV proteins except G where there was a sharp reduction. This is consistent with our other data (Fig. 3a) demonstrating that ProK treatment is effectively removing the extracellular domain of the VSV G protein. Removal of the extracellular domain was confirmed by determining the distribution of the spectra and peptides derived from the G protein (Fig. 4b). The mean number of spectra associated with the transmembrane domain and cytoplasmic tail of the G protein were unchanged between whole and ProK treated virions, while ProK treatment decreased the number of spectra associated with the extracellular domain of G. In looking at the distribution of the detected peptides in the G protein, ProK treatment completely eliminated peptides associated with the N-terminal portion of the extracellular domain while a few were still detected in the more C-terminal portion of the domain, suggesting this region has a slight degree of resistance to proteinase cleavage, although the decrease in spectral counts suggests the majority the G proteins were cleaved.

While MS analysis of unlabeled proteins cannot be used to make quantitative comparisons of protein abundance, spectral counts or measures derived from spectral counts (including emPAI values shown in Table S1), can be used to make semi-quantitative comparisons [61]. Based on spectral counts, few if any of the host proteins come close to matching the viral proteins in abundance within the virion. Only one host protein, low-density lipoprotein receptor-related protein 1 (Lrp1), had a mean of more than 100 total spectra per technical replicate in untreated whole virions, while only 17 (9.4%) had a mean of 20 or more (Table S1). No proteins found only in ProK treated virions had a mean spectral count of more than 20, indicating that these proteins are present in the virions in low abundance.

### Host protein localization within VSV virions

As discussed in the previous section, proteins not associated with the interior of the virion, including proteins embedded in the host derived viral envelope, can be identified by their absence in ProK treated samples or by a size shift upon ProK treatment. Here we wanted to examine the localization within the VSV virion of host proteins that did not display any of these patterns (i.e. proteins that do not appear to be associated with the viral envelope). Five of the chosen proteins were found in both whole and ProK treated virion samples and had similar spectral counts in both sample types (Table S1). We also chose two proteins, proto-oncogene tyrosine-protein kinase Yes (Yes1) and protein tyrosine phosphatase type IVA 2 (Ptp4a2; also called Prl2), detected in ProK treated virions but not whole virions, as proteins found in the treated samples would also be expected to be found in the whole virions from which they were derived (Fig. 2). To identify proteins associated with VSV RNP complexes, we also isolated and analyzed viral RNP complexes from a separate preparation of purified VSV virions by treating them with detergent and salt to release the nucleocapsid from the envelope and M protein while maintaining its association with the L/P polymerase complex.

**Figure 5:**
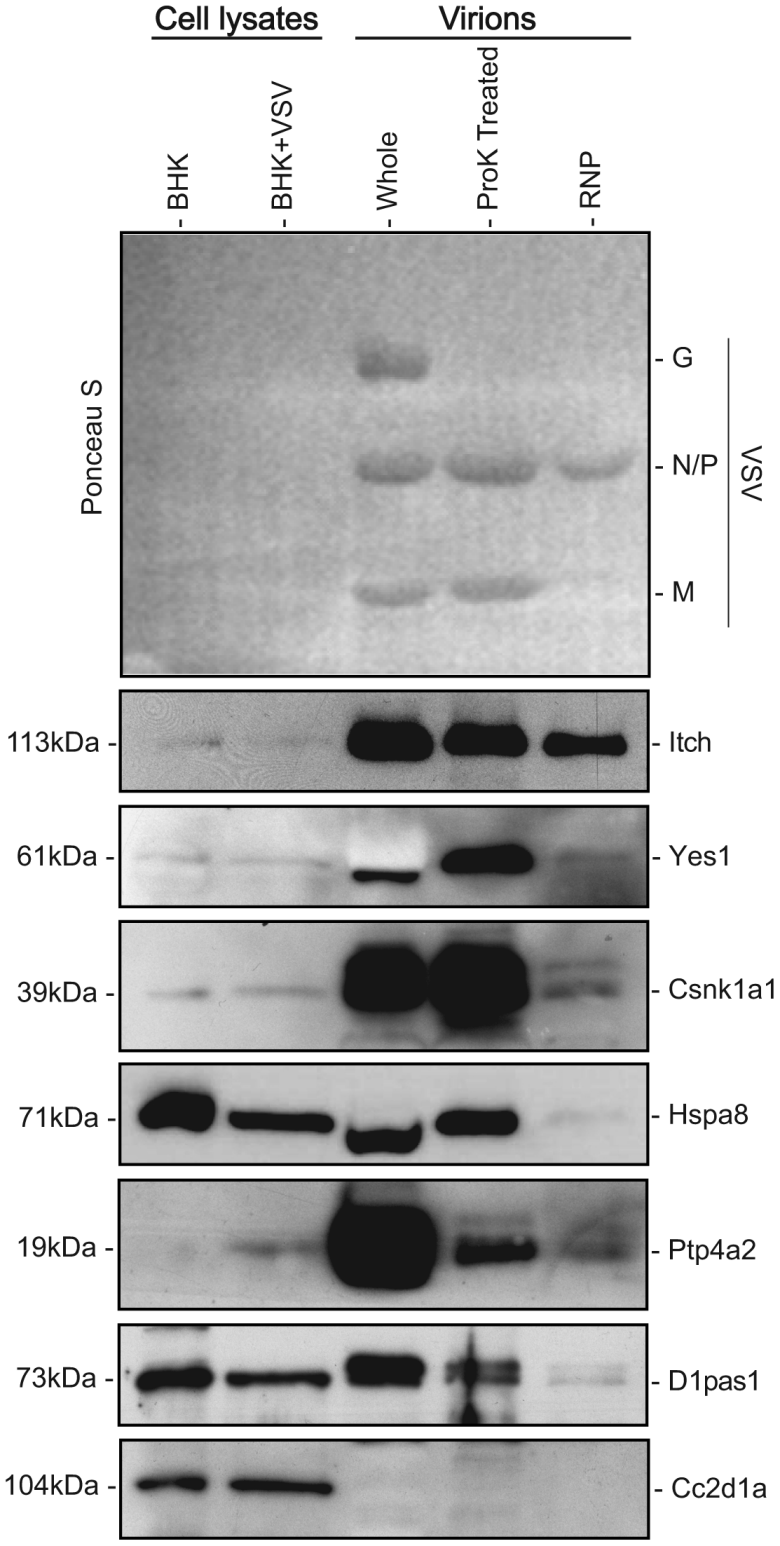
Confirmation of host protein incorporation in virion preparations. Protein lysates from uninfected (BHK) and VSV infected (BHK+VSV) BHK-21 cells were separated by SDS-PAGE along with total protein from whole virions, proteinase K (ProK) treated virions, and viral ribonucleoprotein complexes (RNP). Loading of the virion samples was confirmed by Ponceau S staining of the membrane. The position of the viral glycoprotein (G), nucleocapsid protein (N), phosphoprotein (P), and matrix protein (M) is indicated. Western blots using primary antibodies against coiled-coil and C2 domain-containing protein 1A (Cc2d1a), protein tyrosine phosphatase type IVA 2 (Ptp4a2), putative ATP-dependent RNA helicase Pl10 (D1pas1), proto-oncogene tyrosine-protein kinase Yes (Yes1), casein kinase I isoform alpha (Csnk1a1), heat shock cognate 71 kDa protein kDa protein (Hspa8) and E3 ubiquitin-protein ligase Itchy (ITCH), were conducted as indicated to determine the presence of the host proteins. Approximate molecular mass of the proteins is given on the left.

Because protein quantities were chosen so that the amount of N and P protein was approximately equal for all virion samples (Fig. 5), the host protein associated with whole virions that was retained in the treated samples could be estimated. Based on this, proteins were determined to be associated with the viral RNP complex, associated primarily with the interior of the virion but not the RNP or associated primarily with the exterior of the virion. Most proteins were barely detectable in the RNP complexes, suggesting they do not associate with the viral RNP or that the association was disrupted under the conditions used for RNP isolation. Only for E3 ubiquitin-protein ligase Itchy (ITCH) were substantial amounts of protein detected in RNP. The M protein of VSV, as well as other rhabdoviruses, contains a late domain (L-domain) including a proline rich PPxY motif known to bind WW domains found in a number of cellular proteins [62]. VSV M protein has been shown to bind the WW domain of Nedd4 (also detected in this analysis) [63], and can presumably also bind other HECT domain-containing E3 ubiquitin ligases, including ITCH, as has been reported for other viruses with L-domains including PPxY motifs [64]. However, given that ITCH is present in RNP complexes at levels that nearly equal the other two sample types, while M protein is sharply reduced, it seems likely that ITCH may also interact with one of the RNP proteins (N, P or L). Three of the tested proteins, Yes1, casein kinase I isoform alpha (Csnk1a1) and heat shock cognate 71 kDa protein (Hspa8; also known as Hsc70), while barely detectable in RNP, were found at similar levels in whole and ProK treated virions, indicated they are primarily associated with the interior of the virions. In contrast, levels of Ptp4a2 and putative ATP-dependent RNA helicase Pl10 (D1pas1; also known as Ddx3y), dropped sharply in the ProK and RNP samples indicating they are not primarily located in the interior of the virion. One protein, coiled-coil and C2 domain-containing protein 1A (Cc2d1a), identified by MS could not be detected by WB, indicating either protein levels below the threshold of detection, a misidentification of the protein based on homology, or a false-positive identification. The latter seems the least likely as the protein was identified in both sample types, with at least 6 consecutive amino acids matched in manual inspection.

Of the 6 proteins that could be detected by western blot, only 4 were present at similar levels in whole and ProK treated virions, despite similar spectral counts in both sample types and the absence of a size shift. Therefore, while these characteristics may suggest a protein associated with the interior of the virion, localization should be independently confirmed.

### Characterization of identified proteins

**Figure 6:**
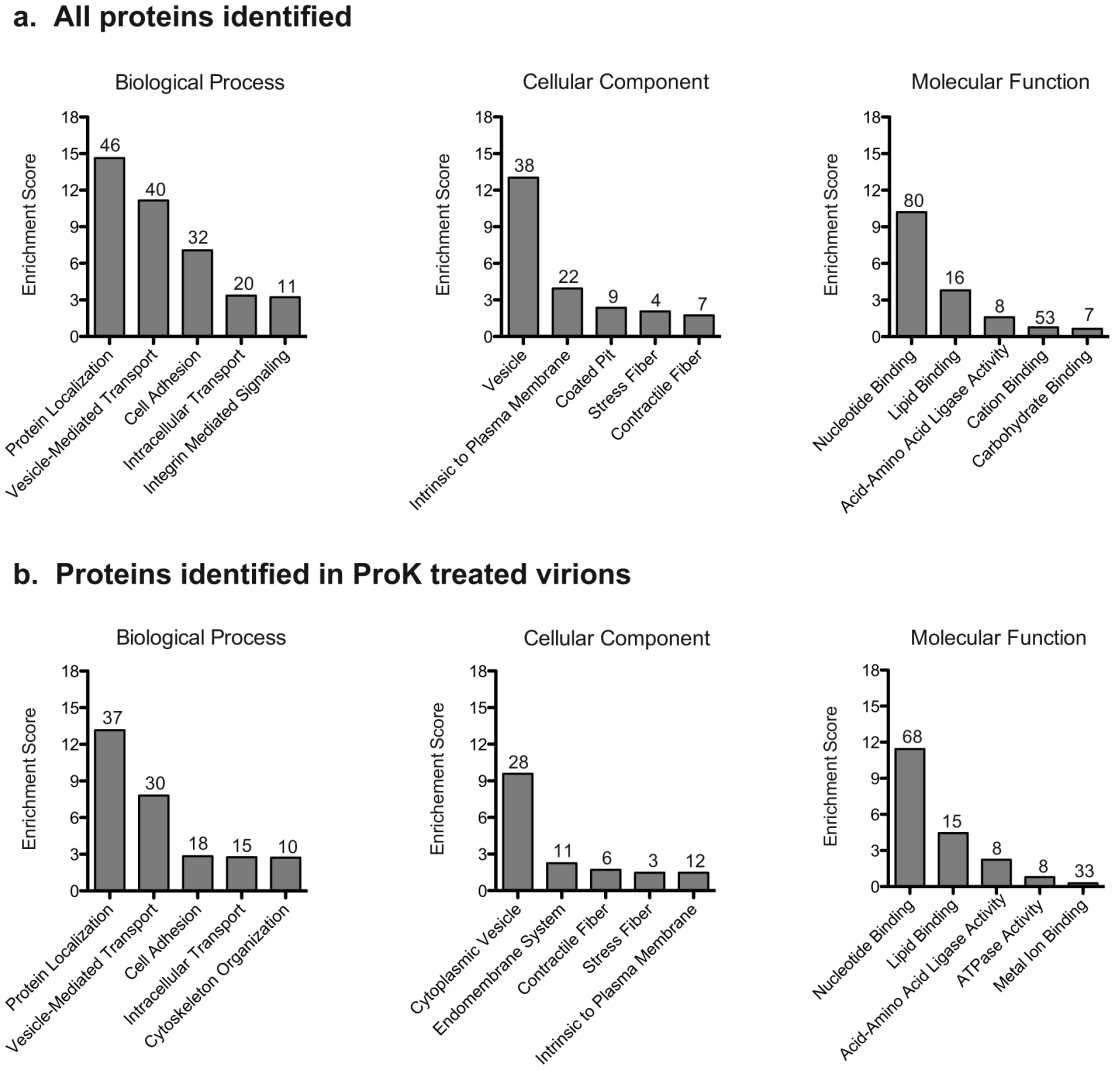
Functional enrichment analysis. Enrichment analysis and clustering based on gene ontology terms was conducted for (a) all proteins identified and (b) proteins identified in the proteinase K (ProK) treated virions, as described in the materials in methods. The five clusters in each database with the highest enrichment score are depicted here. Numbers above the bars indicate the number of identified proteins associated with each cluster.

To obtain an overview of the types of proteins most commonly associated with purified VSV virions, an enrichment analysis was conducted using gene ontology terms. This analysis was done for all three GO databases (biological process, cellular component and molecular function), first using all the host proteins found in our analysis then looking specifically at proteins associated with ProK treated virions (Fig. 6), as this is the highest purity sample and excludes proteins found exclusively on the exterior of the virion. Related enriched terms were then clustered together to give an overall enrichment score. The most highly enriched clusters, when looking at all the proteins, were vesicles and vesicle mediated transport, protein localization and nucleotide binding (Fig. 6). These were also the most highly enriched clusters when looking specifically at proteins identified in ProK treated virions. The cluster cell adhesion was also relatively highly enriched when looking at all proteins, but less so in ProK treated virions as would be expected.

Enrichment of proteins involved in vesicles and vesicle mediated transport, protein localization and cytoskeletal organization is consistent with known features of VSV assembly and budding. More than one third (88/257) of the proteins identified are represented in at least one of these 4 clusters, indicating many virion-incorporated host proteins are likely involved in these functions. Transport of the VSV nucleocapsid to the site of budding has been shown to be dependent on microtubules [65] and is an important step in VSV assembly. Furthermore, as seen for many enveloped viruses (reviewed in [66]), VSV budding appears to use the host proteins involved in multivesicular body (MVB) formation which are relocated from endosomal membranes to the plasma membrane in an M protein dependent manner (reviewed in [67]). However, the budding of VSV and related viruses may still have some unique features. While the specific members of the MVB pathway utilized by VSV for its budding are not clear, it is known that, unlike what has been observed for many other viruses, Tsg101 is not essential for the budding of VSV and rabies virus [68], while contradictory reports exist regarding the importance of Vps4a [68], [69]. In contrast, the host ubiquitin-proteasome system does appear to be essential [63], [69].

The lipid composition of the VSV envelope is consistent with that of its host cell, although levels of cholesterol and sphingomyelin are elevated [48], indicating that unlike some other viruses such as influenza virus and HIV-1 [70], [71], [72], VSV does not bud through host membrane regions enriched in lipid rafts. Instead, VSV G and M proteins appear to initially localize to separate microdomains of the host plasma membrane but then either merge or cluster together with each other and host protein containing microdomains at the site of virus budding [73]. Therefore, VSV readily incorporates the membrane proteins of its host. In pseudotyping experiments using both mixed virus infections and expression of viral or host proteins from VSV recombinants, non-VSV proteins can make up a significant portion of the protein in the VSV envelope, with incorporation levels up to 31% of that seen for VSV G protein reported [74], [75]. Consistent with these observations, 46% (118/257) of proteins identified in our study are associated with the cellular component GO term “plasma membrane”, suggesting they were acquired along with the envelope. The tendency of VSV to indiscriminately acquire relatively large numbers of host membrane proteins has the potential to be exploited to help fine tune therapeutic vectors through the choice of cell line used to generate the viruses. For example, growing oncolytic VSVs in a cell line naturally expressing or engineered to express high levels of complement control protein may improve efficacy by slowing the rate of clearance by the host immune system following administration.

While many of the proteins identified in VSV virions appear to be associated with viral assembly, budding or the host-derived viral envelope, they may also have additional functions that affect virus replication. Furthermore, proteins were also identified that do not have known associations with these functions. Our study provides a valuable inventory of virion-associated host proteins for further investigation into their potential roles in VSV replication cycle, pathogenesis, and immunoreactivity.

## Supporting Information

Table S1 **Cellular proteins identified in whole and ProK treated VSV virions following 1-D SDS-PAGE and UPLC-MS/MS.**
(XLSX)
